# Meta-analysis Using Flexible Random-effects Distribution Models

**DOI:** 10.2188/jea.JE20200376

**Published:** 2022-10-05

**Authors:** Hisashi Noma, Kengo Nagashima, Shogo Kato, Satoshi Teramukai, Toshi A. Furukawa

**Affiliations:** 1 Department of Data Science, The Institute of Statistical Mathematics, Tokyo, Japan; 2 Research Center for Medical and Health Data Science, The Institute of Statistical Mathematics, Tokyo, Japan; 3 Department of Statistical Inference and Mathematics, The Institute of Statistical Mathematics, Tokyo, Japan; 4 Department of Biostatistics, Graduate School of Medical Science, Kyoto Prefectural University of Medicine, Kyoto, Japan; 5 Departments of Health Promotion and Human Behavior, Kyoto University Graduate School of Medicine/School of Public Health, Kyoto, Japan

**Keywords:** meta-analysis, random-effects model, flexible probability distribution, model inadequacy, predictive distribution

## Abstract

**Background:**

In meta-analysis, the normal distribution assumption has been adopted in most systematic reviews of random-effects distribution models due to its computational and conceptual simplicity. However, this restrictive model assumption is possibly unsuitable and might have serious influences in practices.

**Methods:**

We provide two examples of real-world evidence that clearly show that the normal distribution assumption is explicitly unsuitable. We propose new random-effects meta-analysis methods using five flexible random-effects distribution models that can flexibly regulate skewness, kurtosis and tailweight: skew normal distribution, skew *t*-distribution, asymmetric Subbotin distribution, Jones–Faddy distribution, and sinh–arcsinh distribution. We also developed a statistical package, **flexmeta**, that can easily perform these methods.

**Results:**

Using the flexible random-effects distribution models, the results of the two meta-analyses were markedly altered, potentially influencing the overall conclusions of these systematic reviews.

**Conclusion:**

The restrictive normal distribution assumption in the random-effects model can yield misleading conclusions. The proposed flexible methods can provide more precise conclusions in systematic reviews.

## INTRODUCTION

In meta-analysis in medical studies, random-effects models have been the primary statistical tools for quantitative evaluation of treatment effects that account for between-studies heterogeneity.^1^^,^^2^ Conventionally, the normal distribution assumption has been adopted in most systematic reviews due to its computational and conceptual simplicity.^2^^,^^3^ However, the shape of the random-effects distribution reflects how the treatment effects parameters (eg, mean difference, log relative risk) are distributed in the target population, and are directly associated with the fundamental heterogeneity of treatment effects. If the normal distribution assumption diverges drastically from the true heterogeneous structure, the overall results of the meta-analyses may be misleading. In addition, in recent studies, prediction intervals have been gaining prominence in meta-analyses as a means to quantify heterogeneity and effectiveness in real-world uses of the treatment.^4^^,^^5^ Because the prediction interval is constructed by the estimated random-effects distribution, it should be directly influenced by the form of the distribution assumptions.

Several papers have discussed the flawed uses of the normal distribution model in meta-analyses^6^^–^^11^ involving alternative effective modelling strategy to handle outliers. Baker and Jackson^9^ discussed the use of several non-normal random-effects distributions to handle outliers in meta-analyses; for example, *t*-distribution and Subbotin distribution. Lee and Thompson^6^ also discussed Bayesian approaches using flexible skewed normal and *t*-distributions of Fernandez and Steel.^12^ In addition, Baker and Jackson^10^ proposed direct marginal modelling approaches of the study-specific true underling effects that can express skewed distributions and do not require numerical integrations for the likelihood-based inferences. However, to date there are still limited methods to address this relevant issue, especially to handle skewness, kurtosis, and tailweight of the random-effects distributions flexibly. Also, there are only limited useful statistical packages that can be handled by non-statisticians; for example, although the R package metaplus^13^ can handle several non-normal distributions, it is only limited to *t*-distribution and normal mixture distribution. Particularly, the well-established Azzalini’s skew normal distribution and related families^14^ have not been discussed in the meta-analysis methodology.

In this article, we propose random-effects meta-analysis methods with flexible distribution models that can flexibly express skewness, kurtosis, and tailweight: (1) skew normal distribution,^14^^,^^15^ (2) skew *t*-distribution,^14^^,^^16^ (3) asymmetric Subbotin distribution,^14^^,^^17^ (4) Jones–Faddy distribution,^18^ and (5) sinh–arcsinh distribution.^19^ Via application of these five flexible random-effects distribution models to two recently published systematic reviews,^20^^,^^21^ we will demonstrate that the overall conclusions and interpretations of meta-analyses can be dramatically altered if the normal distribution assumption is not suitable. In addition, we provide a new R package, **flexmeta**, that can perform meta-analysis with simple code using the flexible random-effects distributions. We will explicitly show that the implicit uses of the normal distribution assumption might yield misleading results, and that our flexible alternative distributions may provide more valid conclusions for health technology assessments and policy making.

## METHODS

### Descriptions of two motivating meta-analyses

**Figure 1.**
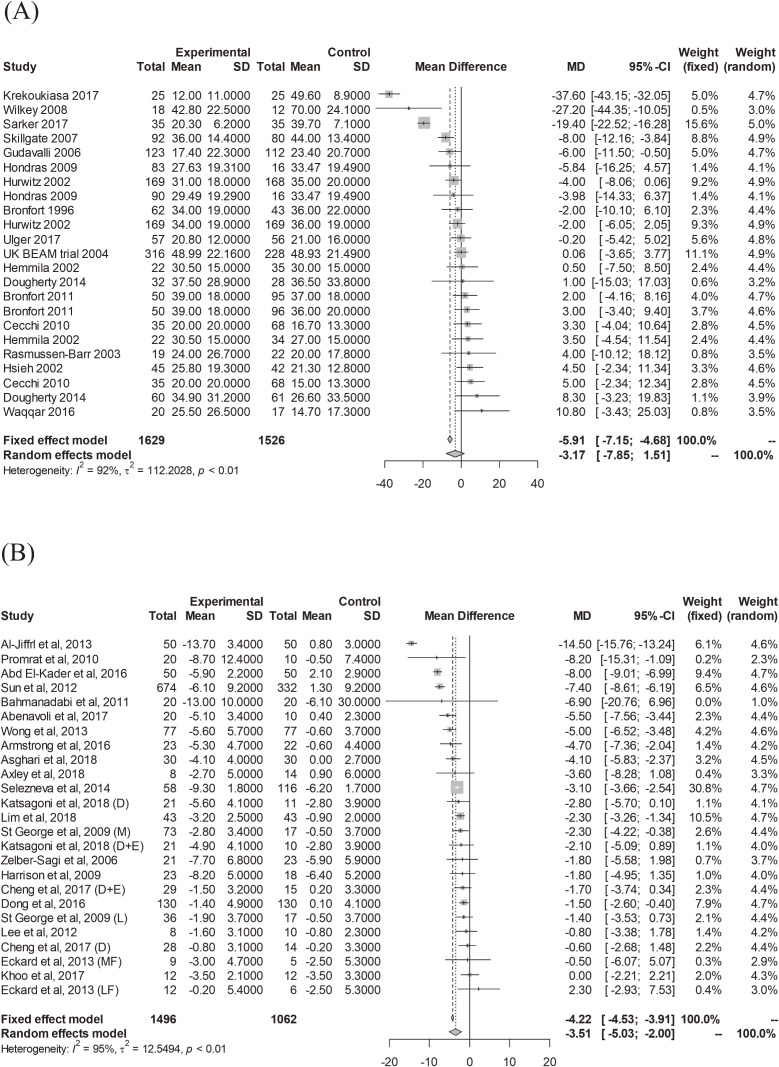
Forest plots for the two motivating examples: (A) meta-analysis of chronic low back pain^20^ and (B) meta-analysis of nonalcoholic fatty liver disease^21^

We searched for recently published systematic reviews in leading medical journals (eg, *BMJ*, *JAMA*), and found two examples^20^^,^^21^ that clearly demonstrated the unsuitability of the normal distribution assumption. The first example is a meta-analysis by Rubinstein et al^20^ assessing the benefits and harms of spinal manipulative therapy (SMT) for the treatment of chronic lower back pain. In Figure 1A, we present a forest plot of their meta-analysis of 23 randomized controlled trials assessing the reduction of pain at 1 month (0–100; 0 = no pain, 100 = maximum pain) for SMT (*N* = 1,629) versus recommended therapies (*N* = 1,526). The effect measure was the mean difference (MD). Using the ordinary random-effects meta-analysis method based on the normal random-effects distribution, we identified a substantial heterogeneity of the treatment effects, *I*^2^ = 92%, *τ*^2^ = 112.20 (*P* < 0.01; Cochrane’s Q-test). The between-studies heterogeneity should be addressed in synthesis analysis. However, most of the MD estimates fell within relatively narrow range around the mean, although a small number exhibited larger effect sizes. This might imply that the true MD distribution is a skewed, heavy-tailed, and sharp distribution. Although the average MD was estimated as −3.17 (95% confidence interval [CI], −7.85 to 1.51) using the DerSimonian-Laird method,^3^ the point and interval estimates depend on the normal distribution assumption.

The second example is a meta-analysis by Koutoukidis et al^21^ aimed at estimating the association of weight loss interventions with biomarkers of liver disease in nonalcoholic fatty liver disease. In Figure 1B, we also present a forest plot of their meta-analysis of 25 randomized controlled trials that assess the weight loss (kg) for more-intensive weight loss interventions (*N* = 1,496) versus no or lower-intensity weight loss interventions (*N* = 1,062). Again, the effect measure was the MD, and we identified a substantial heterogeneity of the treatment effects: *I*^2^ = 95%, *τ*^2^ = 12.45 (*P* < 0.01; Cochrane’s Q-test). In this case, the MD estimates were not symmetrically distributed, and a certain number of trials exhibited a larger intervention effect than the average MD of −3.51 (95% CI, −5.03 to −2.00). Thus, the true MD distribution would be a skewed, heavy-tailed distribution. In particular, in predicting the intervention effect of a future trial, the normal distribution model would not suitably fit this dataset. Although the ordinary 95% Higgins–Thompson–Spiegelhalter (HTS) prediction interval^5^ was (−11.02 to 3.99), it might not express the true nature of the intervention effects in the target population.

### The flexible random-effects distribution models

To address the restriction problem of the normal distribution, we propose random-effects meta-analysis methods using five flexible random-effects distribution models based on Bayesian methodology. For the notation, we consider that there are *K* studies to be synthesized, and that *Y_i_* (*i* = 1, 2, …, *K*) is the estimated treatment effect measure in the *i*th study, eg, mean difference, odds ratio, and hazard ratio; the ratio measures are typically transformed to logarithmic scales. The random-effects models considered here^2^^,^^3^ are then defined as

$$Y_{i}∼N(\theta_{i},\sigma_{i}^{2})$$ (*)

$$\theta_{i}∼F(\theta )$$

where *θ_i_* is the true effect size of the *i*th study, and $\sigma_{i}^{2}$ is the within-studies variance, which is usually assumed to be known and fixed to valid estimates. Note that the normal likelihood model adopts a large sample approximation, and it is possibly a substantial limitation of this model; however, the source codes for the **flexmeta** package are available at the Github site (https://github.com/nomahi/flexmeta), and users can customize the likelihood models freely. Also, *F*(*θ*) corresponds to the random-effects distribution that expresses the heterogeneous probability distribution of *θ_i_*. For the conventional normal-normal random-effects model, *F*(*θ*) corresponds to a normal distribution. The predictive interval for future study^4^ is substantially constructed based on the random-effects distribution *F*(*θ*). To overcome the limitations on the expressive ability of the normal distribution, our proposal is to adopt alternative flexible probability distributions.

**Figure 2.**
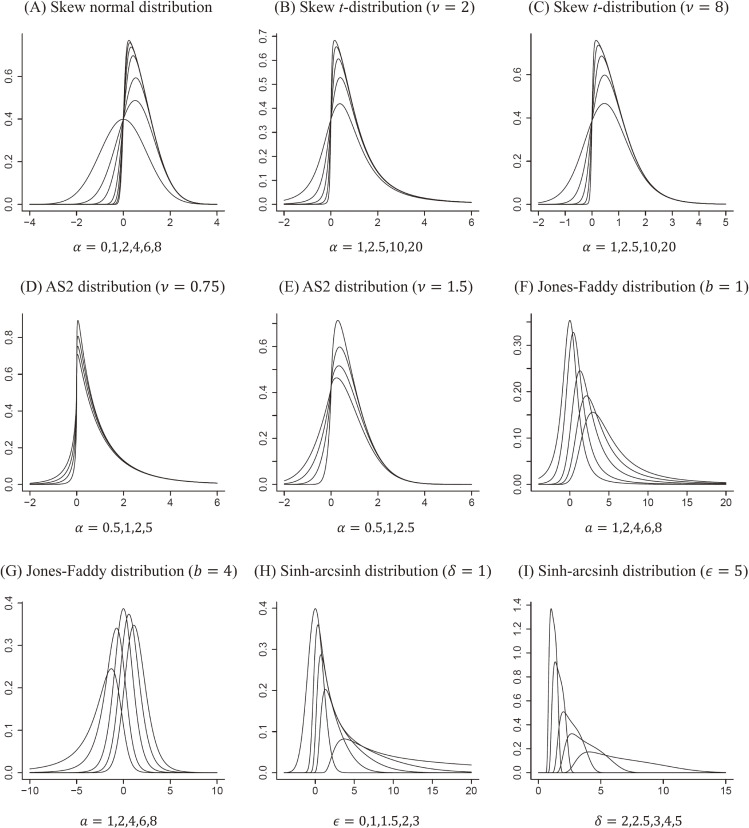
Flexible models for the random-effects distribution: (A) skew normal distribution; (B), (C) skew *t*-distribution; (D), (E) AS2 distribution; (F), (G) Jones–Faddy distribution; (H), (I) sinh–arcsinh distribution

Currently, due to developments in statistical distribution theory, various flexible probability distributions are available.^14^ Here, we chose five representatives of the newest recently developed distributions. In Figure 2, we present some examples of these five distributions and their ability to express various shapes. Their mathematical details are presented in eAppendix 1 at *Journal of Epidemiology* online; here, we describe them in a non-technical manner.

### Skew normal distribution SN(ξ, ω, α)

The skew normal distribution^14^^,^^15^ is a generalized version of the conventional normal distribution that allows for skewness. *ξ* is the location parameter that regulates the center location, and *ω* is the scale parameter that regulates the dispersion of the distribution; we use these notations similarly for the following four distributions as well; in the graphical displays in Figure 2, we set these parameters to *ξ* = 0 and *ω* = 1, consistently. Also, *α* is the skewness parameter that adjusts the skewness, and the distribution is positively (negatively) skewed for *α* > 0 (*α* < 0). When *α* = 0, it accords to a normal distribution N(*ξ*, *ω*). In Figure 2A, we present probability density functions of the skew normal distribution with *α* = 0, 1, 2, 4, 6, 8. It can flexibly express skew-shaped distributions, but has limitations in expressing kurtosis and tailweight.

### Skew t-distribution ST(ξ, ω, ν, α)

The skew *t*-distribution^16^ is also a generalized version of the conventional Student *t*-distribution that allows for skewness. The *t*-distribution can express heavy tailweight and kurtosis relative to the normal distribution via varying the degree of freedom *ν* (> 0). In this case, *α* is the skewness parameter; the distribution is positively (negatively) skewed for *α* > 0 (*α* < 0). Also, for *α* = 0, it accords to the ordinary *t*-distribution. In Figure 2B and Figure 2C, we present the skew *t*-distribution with *α* = 1, 2, 4, 5, 10, 20 with *ν* = 2, 8. The skew *t*-distribution can express flexible shapes by controlling the degree of freedom, relative to the skew normal distribution, especially for kurtosis and tailweight.

### Asymmetric Subbotin distribution (Type II) AS2(ξ, ω, ν, α)

Subbotin^22^ proposed a symmetric probability distribution that can flexibly regulate the kurtosis and tail thickness; it involves a double exponential and trapezoidal-shaped distributions as special cases. Baker and Jackson^9^ proposed to use the Subbotin distribution for meta-analyses. An extended version of this distribution is the asymmetric Subbotin distribution of type II (AS2).^14^^,^^17^ We present some examples of the AS2 distribution in Figure 2D and Figure 2E, which clearly display its flexible expression ability. *α* is the skewness parameter, and *ν* (> 0) is the degree of freedom that regulates the kurtosis. The distribution is positively (negatively) skewed for *α* > 0 (*α* < 0), and more kurtosed for smaller *ν*.

### Jones–Faddy distribution JF(ξ, ω, a, b)

Jones and Faddy^18^ proposed another skewed version of the *t*-distribution. The Jones–Faddy distribution regulates the skewness and kurtosis by two parameters, *a* (> 0) and *b* (> 0); *ξ* and *ω* are the location and scale parameters. Some examples are provided Figure 2F and Figure 2G, some examples are provided. This distribution is positively (negatively) skewed for *a* > *b* (*a* < *b*). Also, it reduces to *t*-distribution for *a* = *b*, with *a* + *b* degrees of freedom. It can also flexible express various skewed, sharp, and heavy-tailed distributions by regulating the four parameters.

### Sinh–arcsinh distribution SAS(ξ, ω, δ, ϵ )

Jones and Pewsey^19^ proposed a flexible unimodal four-parameter distribution that is induced by sinh–arcsinh (SAS) transformation. The SAS distribution can express symmetric and skewed shapes with heavy and light tailweight. In Figure 2H and Figure 2I, several examples are presented. *ξ* and *ω* are the location and scale parameters, *δ* (> 0) is the kurtosis parameter, and ϵ is the skewness parameter. This distribution is positively (negatively) skewed for ϵ>0 ( ϵ<0 ). The kurtosis is regulated by *δ*. It can express various skew *t*-distributions with quite sharp and gently sloped shapes with various degrees of skewness.

### Methods for the treatment effect estimation and prediction

For the random-effects model (*), we can adopt the flexible distribution models for the random-effects distribution *F*(*θ*). The average treatment effect can be addressed as the mean *μ* of *F*(*θ*). As in the conventional DerSimonian-Laird-type normal-normal model, the parameters of *F*(*θ*) can be estimated by frequentist methods (eg, the maximum likelihood estimation), but in many cases, they require complex numerical integrations; the computations of CIs and *P*-values also have computational difficulties. Besides, through Bayesian approaches, we can compute posterior distributions of the mean parameter using a unified Markov Chain Monte Carlo (MCMC) framework.^23^^,^^24^ In addition, under the Bayesian framework, we can directly assess the predictive distribution of the treatment effect for a future study by the posterior predictive distribution.^5^ When using the flexible parametric distributions, we can directly assess the nature and degree of heterogeneity using the predictive distribution. The variance of these distributions can be similarly defined for these distributions, but might not be properly interpreted as a dispersion parameter for skewed distributions. For these flexible skewed distributions, the predictive distributions can be directly used as a heterogeneity measure. Also, if we assume a non-informative prior distribution, the posterior inference can be substantially equivalent to the frequentist inference. We adopted vague priors for all model parameters in the random-effects distribution models. For comparisons of competing models, we can use model assessment criteria of Bayesian statistics (eg, the deviation information criterion [DIC]).^25^ These computations can be easily performed by simple commands using the R package **flexmeta** (available at https://github.com/nomahi/flexmeta). The methodological details for the Bayesian modelling are presented in eAppendix 1.

### Applications to the two meta-analyses

We applied the flexible random-effects models to the two meta-analysis datasets described above. As reference methods, we also conducted the same analyses using the normal and *t*-distribution models. We used R ver. 3.5.1 (R Foundation for Statistical Computing, Vienna, Austria) and the **flexmeta** package for the statistical analyses; to implement MCMC, we used RStan ver. 2.19.2.^26^ After 10,000 warm-ups, 250,000 samples were used for the posterior inferences and prediction. The 95% credible intervals (CrI) and predictive intervals (PI) were calculated using the posterior samples of the mean of *F*(*θ*) and the predictive distribution of the effect of a future study *θ_new_* ∼ *F*(*θ*) from MCMC. To evaluate the impacts of adopting the flexible distribution models rather than the ordinary normal distribution, we present graphical displays of the posterior and predictive distributions. In addition, we assessed model adequacies by DIC.

## RESULTS

**Figure 3.**
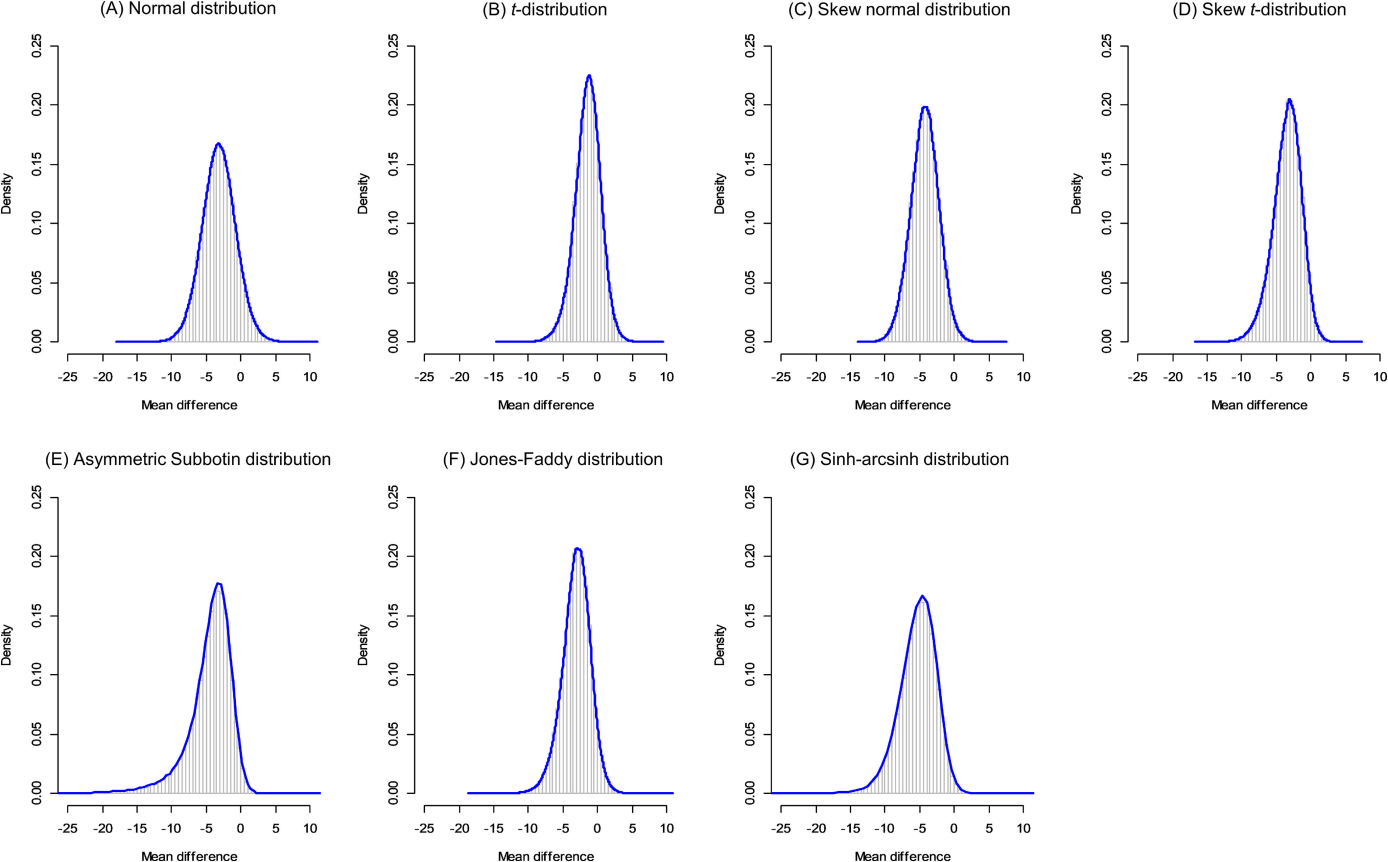
Posterior distributions for *μ* of the meta-analysis of chronic low back pain^20^ using seven random-effects distribution models

In Table 1A, we present the summary of the posterior distributions for the mean *μ* of the random-effects distributions. For the first example, the meta-analysis of chronic lower back pain, the posterior summary of the normal distribution is similar to the results of the conventional method, and the overall MD is −3.17 (95% CrI, −8.02 to 1.73). The posterior means and 95% CrI of *μ* were quite different. In Figure 3, we present graphical displays of the 250,000 posterior samples of *μ*. All of the estimated posterior distributions using the five flexible random-effects distribution models indicated skewed and sharp-shaped distributions; it was sharper even for the *t*-distribution. DIC comparisons suggested that the best-fitting model was the AS2 distribution (DIC = 139.03); the SAS distribution was comparable to it (DIC = 139.97). Both of these distributions yielded larger MD estimates: −3.99 (95% CrI, −9.47 to −0.10) and −5.33 (95% CrI, −11.37 to −0.94), respectively. In addition, the posterior probabilities that *μ* is smaller than 0 were 0.98 and 0.99, respectively, whereas that of the normal random-effects distribution model was 0.90. In the original paper by Rubinstein et al^20^ the overall MD test was not statistically significant at the 5% level. However, the overall results were clearly altered by adopting the skewed flexible distribution models, which strongly indicated that the true effect sizes would lie within a narrower range and would be skew distributed. The overall conclusion for the overall MD could be changed using the flexible models. Also, we present a summary of the predictive distribution of this example in Table 2A. These results also indicated that the predictive distribution would be strongly skewed. Note that the random-effects distributions with similar DICs had quite different posterior distributions (eg, SN, AS2, JF, and SAS), DIC is not an absolute measure to select “correct” statistical models and such results can commonly occur. There are many other useful measures for model selections,^27^ and users can adopt other criteria via customizing the source codes of **flexmeta** available at the Github site.

**Table 1.** Summary of the posterior distributions for the mean *μ* of the random-effects distribution^20^^,^^21^

Random-effects distribution	Mean	SD	95% CrI	Pr(*μ* < 0)	DIC
(A) Meta-analysis of the treatment of chronic low back pain					
Normal distribution	−3.17	2.47	(−8.02, 1.73)	0.90	145.62
*t*-distribution	−1.43	1.91	(−5.47, 2.14)	0.78	143.54
Skew normal distribution	−4.27	2.05	(−8.37, −0.32)	0.98	141.91
Skew *t*-distribution	−3.47	2.09	(−8.04, 0.19)	0.97	140.50
AS2 distribution	−3.99	2.39	(−9.47, −0.10)	0.98	139.03
Jones–Faddy distribution	−3.09	2.05	(−7.49, 0.65)	0.95	141.94
Sinh–arcsinh distribution	−5.33	2.91	(−11.37, −0.94)	0.99	139.97
(B) Meta-analysis of the treatment of nonalcoholic fatty liver disease					
Normal distribution	−3.52	0.77	(−5.04, −1.98)	1.00	99.27
*t*-distribution	−3.06	0.66	(−4.43, −1.81)	1.00	97.95
Skew normal distribution	−3.83	0.69	(−5.29, −2.55)	1.00	96.52
Skew *t*-distribution	−3.61	0.69	(−5.14, −2.40)	1.00	95.44
AS2 distribution	−3.78	0.83	(−5.72, −2.44)	1.00	94.72
Jones–Faddy distribution	−3.49	0.68	(−4.93, −2.23)	1.00	97.02
Sinh–arcsinh distribution	−4.24	0.86	(−6.03, −2.75)	1.00	96.70

AS2, Asymmetric Subbotin distribution (Type II); CrI, credible interval, DIC, deviance information criterion.

**Table 2.** Summary of the predictive distributions for the two meta-analyses^20^^,^^21^

Random-effects distribution	Mean	SD	95% PI	Pr(*θ_new_* < 0)
(A) Meta-analysis for the treatment of chronic low back pain				
Normal distribution	−3.17	11.33	(−25.62, 19.29)	0.61
*t*-distribution	−1.43	9.69	(−20.46, 17.35)	0.58
Skew normal distribution	−4.26	9.43	(−26.42, 9.92)	0.63
Skew *t*-distribution	−3.47	9.60	(−26.85, 8.51)	0.59
AS2 distribution	−4.22	10.56	(−32.22, 6.81)	0.58
Jones–Faddy distribution	−3.09	9.65	(−24.65, 11.22)	0.59
Sinh–arcsinh distribution	−5.37	11.80	(−33.51, 7.67)	0.60
(B) Meta-analysis for the treatment of nonalcoholic fatty liver disease				
Normal distribution	−3.52	3.63	(−10.71, 3.68)	0.84
*t*-distribution	−3.06	3.37	(−9.77, 3.51)	0.86
Skew normal distribution	−3.83	3.18	(−11.31, 0.93)	0.93
Skew *t*-distribution	−3.61	3.23	(−11.53, 0.55)	0.95
AS2 distribution	−3.81	3.57	(−12.96, 0.15)	0.97
Jones–Faddy distribution	−3.49	3.20	(−10.78, 1.72)	0.91
Sinh–arcsinh distribution	−4.31	3.72	(−12.78, 0.61)	0.95

AS2, Asymmetric Subbotin distribution (Type II); PI, predictive interval.

**Figure 4.**
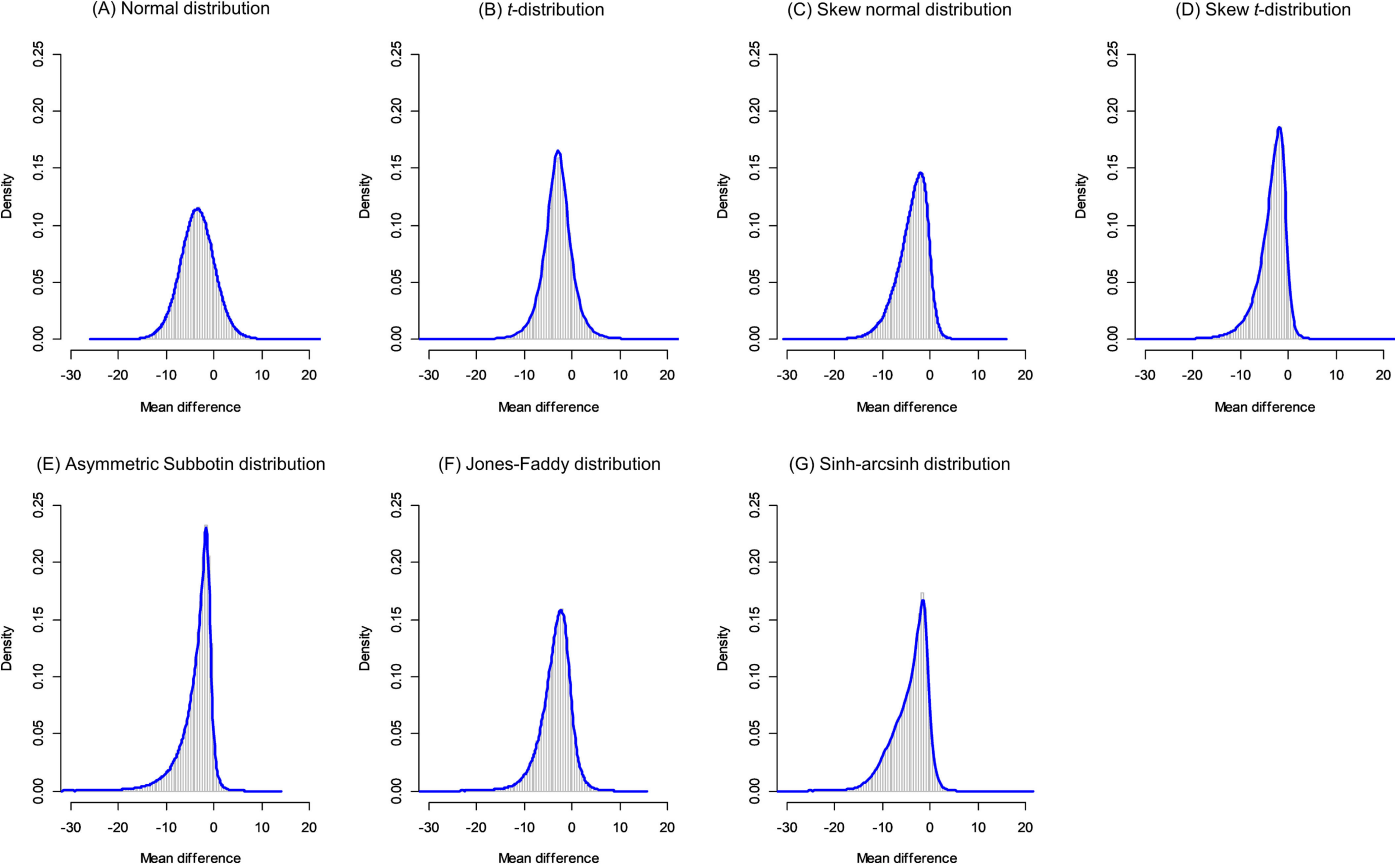
Predictive distributions for the meta-analysis of nonalcoholic fatty liver disease^21^ using seven random-effects distribution models

For the second example, the meta-analysis of non-alcoholic fatty liver disease, we present summaries of the posterior distribution of *μ* and the predictive distribution in Table 1B and Table 2B. For this case, the normal distribution model provides results that are similar to those of the conventional methods. However, DIC comparisons revealed that the normal distribution was the worst-fitted model (DIC = 99.27), whereas AS2 and skew *t*-distribution were the best-fitted (DIC = 94.72 and 95.44, respectively). For the overall MD *μ*, the flexible models exhibited more skewed posterior distributions and larger MD estimates. Further, in Figure 4, we present the predictive distributions of the seven distribution models. We found that all of the flexible distribution models exhibited skewed and sharp-shaped distributions. In particular, the well-fitted AS2 and skew *t*-distributions indicated that the treatment effect in a future study *θ_new_* would lie within a narrower range and would be skew distributed. However, the 95% PI of the normal distribution model was −10.71 to 3.68, those of AS2 and skew *t*-distribution were −12.96 to 0.15 and −11.53 to 0.55, respectively. The posterior probabilities of Pr(*θ_new_* < 0) of the normal distribution model was 0.84, whereas those of AS2 and skew *t*-distribution were 0.97 and 0.95, respectively. Hence, the overall conclusions might be altered. Note that the resultant posterior inferences and predictions depended on the prior distributions (see eAppendix 2), and the sensitivity should be carefully considered in practices.

## DISCUSSION

Conclusions obtained from meta-analyses are widely applied to public health, clinical practice, health technology assessments, and policy-making. If misleading results have been produced by inadequate methods, the impact might be enormous. In this article, we proposed effective methods for meta-analysis using flexible random-effects distribution models, and provided an easily implementable statistical package for these methods. Through illustrative examples, we clearly showed the restrictions of using the conventional normal random-effects distribution model, which may yield misleading conclusions. The flexible random-effects distribution models represent effective tools for preventing such an outcome. Conventionally, these MCMC computations require special software and high-performance computers; to address these obstacles, we developed a user-friendly package, **flexmeta**, which was designed to be easily handled by non-statisticians and is freely available online. The proposed methods and the developed tools would help us to provide precise evidence. At a minimum, we recommend using these methods in sensitivity analyses.

In this study, we adopted five flexible distributions, but other probability distributions exist in statistical theory (eg, see the comprehensive textbook by Azzalini and Capitanio).^14^ Other choices might also be considered, but the five distribution models discussed here have sufficient expressive abilities, and significantly different results would not be obtained by adopting other existing distributions. Another choice would be to adopt nonparametric methods.^23^^,^^24^ However, in meta-analysis in medical studies, the number of studies *K* is usually not large^28^^,^^29^; consequently, nonparametric methods would be unstable in many applications because they require much larger statistical information (parallel to *K*) to conduct valid estimation and prediction. Also, the proposed methods have the same limitations because the flexible random-effects distributions would not be identifiable if the number of studies is small. Especially, when the number of studies is less than 5, even the normal distribution models are difficult to estimate.^30^ This problem would be a substantial limitation of these methods.

In addition, the normal distribution model implicitly involves some relevant hidden assumptions that were well discussed in Jackson and White.^11^ The fully parametric assumptions for a random-effects distribution similarly induces the same limitations, but the flexible alternative distribution assumptions address a relevant limitation “the shape of the normal distribution is assumed (for the random-effects distribution), not just the first two moments”.^11^ Although various methods to overcome the limitations of the normal distribution assumptions have been developed, there are no unique methods that address the all limitations discussed by Jackson and White^11^ uniformly. The proposed methods in this paper would be added as effective tools to address the relevant issue.

As shown by the real data applications, existing meta-analyses may have reached misleading conclusions due to the straightforward uses of the normal random-effects distribution model. Our proposed methods might change the overall conclusions of these meta-analyses, and systematic re-evaluation of existing meta-analyses would be an interesting topic for future studies. In addition, for future systematic reviews, the flexible methods might be used as standard methods to provide accurate conclusions, at least in sensitivity analyses.

## SUPPLEMENTARY MATERIAL

eAppendix 1: Mathematical details of the flexible distributions

eAppendix 2: Methods for the Bayesian modelling
