# Supplementary material for: Meta-analysis Using Flexible Random-effects Distribution Models
Source: J Epidemiol. 2022 Oct 5;32(10):441–8. doi: 10.2188/jea.JE20200376 (PMC9424185; doi:10.2188/jea.JE20200376)
Supplement: Supplementary file 1 [file je-32-441-s001.pdf]

SUPPLEMENTARY DATA for

Meta-analysis using flexible random-effects distribution models

Hisashi Noma[1], Kengo Nagashima[2], Shogo Kato[3], Satoshi Teramukai[4]
and Furukawa, T. A.[5]

[1] *Department of Data Science, The Institute of Statistical Mathematics, Tokyo, Japan*
[2] *Research Center for Medical and Health Data Science, The Institute of Statistical Mathematics, Tokyo, Japan*
[3] *Department of Statistical Inference and Mathematics, The Institute of Statistical Mathematics, Tokyo, Japan*
[4] *Department of Biostatistics, Graduate School of Medical Science, Kyoto Prefectural University of Medicine, Kyoto, Japan*
[5] *Departments of Health Promotion and Human Behavior, Kyoto University Graduate School of Medicine/School of Public Health, Kyoto, Japan*

eAppendix 1: Mathematical details of the flexible distributions

1.1 Skew normal distribution

The skew normal distribution (Azzalini, 1985; Azzalini and Capitanio, 2014) is a generalized version of the conventional normal distribution that allows for skewness. The probability density function is

$$p(\theta|\xi,\omega,\alpha)=\frac{2}{\omega}\phi\left(\frac{\theta-\xi}{\omega}\right)\Phi\left(\frac{\alpha(\theta-\xi)}{\omega}\right)$$

where $\phi(\theta)$ and $\Phi(\theta)$ are, respectively, the probability density and cumulative distribution functions of the standard normal distribution N(0, 1). ξ is the location parameter that regulates the center location, and ω (> 0) is the scale parameter that regulates the dispersion of the distribution. α is the skewness parameter that adjusts the skewness; the distribution is positively (negatively) skewed for $\alpha > 0$ ($\alpha < 0$). When $\alpha = 0$, the skew normal distribution accords with the normal distribution $\mathrm{N}(\xi,\omega)$. The mean and variance of this distribution are

$$\mathrm{E}[\theta]=\xi+\omega b\delta$$

$$\mathrm{V}[\theta]=\omega^2[1-(b\delta)^2]$$

where

$$b = \sqrt{\frac{2}{\pi}}$$

$$\delta = \frac{\alpha}{\sqrt{1 + \alpha^2}}$$

1.2 Skew t-distribution

The skew *t*-distribution (Azzalini and Capitanio, 2003, 2014) is also a generalized version of the conventional Student *t*-distribution that allows for skewness. The probability density function is

$$p(\theta|\xi, \omega, \nu, \alpha) = \frac{2}{\omega} f_t\left(\frac{\theta - \xi}{\omega}\middle|\nu\right) F_t\left(\frac{\alpha(\theta - \xi)}{\omega}\sqrt{\frac{\omega^2(\nu + 1)}{\nu\omega^2 + (\theta - \xi)^2}}\middle|\nu + 1\right)$$

where $f_t(\theta|\nu)$ and $F_t(\theta|\nu)$ are, respectively, the probability density and cumulative distribution functions of the Student *t*-distribution with ν (> 0) degrees of freedom. ξ is the location parameter that regulates the center location, and ω (> 0) is the scale parameter that regulates the dispersion of the distribution. α is the skewness parameter that adjusts the skewness and the distribution is positively (negatively) skewed for $\alpha > 0$ $(\alpha < 0)$. When $\alpha = 0$, this distribution accords with the Student *t*-distribution with ν degrees of freedom. The mean and variance of this distribution are

$$\mathrm{E}[\theta] = \xi + \omega b_\nu \delta$$
$$\mathrm{V}[\theta] = \omega^2 \left[\frac{\nu}{\nu - 2} - (b_\nu \delta)^2\right]$$

where

$$b_\nu = \frac{\sqrt{\nu}\Gamma((\nu - 1)/2)}{\sqrt{\pi}\Gamma(\nu/2)}$$

$$\delta = \frac{\alpha}{\sqrt{1 + \alpha^2}}$$

The skew *t*-distribution can express flexible shapes by controlling the degree of freedom,

compared with skew normal distribution, especially for the kurtosis and tailweight.

1.3 Asymmetric Subbotin distribution

Subbotin (1923) proposed a symmetric probability distribution that can regulate the kurtosis and tail thickness flexibly. The probability density function of the Subbotin distribution with ν (> 0) degrees of freedom is

$$f_S(\theta) = \frac{1}{2\nu^{1/\nu}\Gamma(1+1/\nu)} \exp\left(-\frac{|\theta|^\nu}{\nu}\right)$$

Based on the form of the probability density function, this distribution involves a double exponential and trapezoidal-shaped distributions as special cases. The location and scale can be regulated by linear transmission, and the distribution can flexibly express heavy and light tailweight. The asymmetric Subbotin distribution of type II (AS2) (Azzalini, 1986) is an extended version of this distribution, and the probability density function is

$$p(\theta|\xi,\omega,\nu,\alpha) = \frac{2}{\omega} f_S\left(\frac{\theta-\xi}{\omega}\middle|\nu\right) F_S\left(\frac{\alpha(\theta-\xi)}{\omega}\right)$$

where

$$F_S(\theta) = \Phi\left(\mathrm{sgn}(\theta)\frac{|\theta|^{\nu/2}}{\sqrt{\nu/2}}\right)$$

ξ is the location parameter that regulates the center location, and ω is the scale parameter that regulates the dispersion of the distribution. The distribution is positively (negatively) skewed for $\alpha > 0$ ($\alpha < 0$), and more kurtosed for smaller ν. The mean and variance of this distribution are

$$\mathrm{E}[\theta] = \xi + \mathrm{sgn}(\alpha)\omega C_\nu Q_\nu$$

$$\mathrm{V}[\theta] = \omega^2\left[\frac{\nu^{2/\nu}\Gamma(3/\nu)}{\Gamma(1/\nu)} - (C_\nu Q_\nu)^2\right]$$

where

$$C_\nu = \frac{\nu^{1/\nu}\Gamma(2/\nu)}{\Gamma(1/\nu)}$$

$$Q_\nu = 2F_t\left(\sqrt{4|\alpha|^\nu/\nu}\,\middle|\,4/\nu\right) - 1$$

As shown in Figure 2, the AS2 distribution can express a sharp skew distribution, which can be seen as an asymmetric double exponential distribution. Also, it can express a more rounded shape, like the skew *t*-distribution.

1.4 Jones-Faddy distribution

Jones and Faddy (2003) proposed another skewed version of *t*-distribution, whose probability density function is expressed as

$$p(\theta|\xi,\omega,\nu,\alpha) = \frac{1}{\omega} f_{JF}\left(\frac{\theta-\xi}{\omega}\,\middle|\,a,b\right)$$

where

$$f_{JF}(z|a,b) = C_{a,b}^{-1}\left\{1+\frac{z}{(a+b+z^2)^{1/2}}\right\}^{a+1/2}\left\{1-\frac{z}{(a+b+z^2)^{1/2}}\right\}^{b+1/2}$$

$$C_{a,b} = 2^{a+b-1}B(a,b)(a+b)^{1/2}$$

The Jones–Faddy distribution regulates the skewness and kurtosis through two model parameters a (>0) and b (>0). ξ is the location parameter that regulates the center location, and ω (>0) is the scale parameter. This distribution is positively (negatively) skewed for $a > b$ ($a < b$). Also, it reduces to the *t*-distribution for $a = b$, with $a + b$ degrees of freedom. It can also flexibly express various distributions involving skewed, sharp, and heavy-tailed shapes by regulating the four parameters. The mean and variance are

$$\mathrm{E}[\theta] = \xi + \omega\eta_{a,b}$$

$$\mathrm{V}[\theta] = \omega^2\left[\frac{a+b}{4}\frac{(a-b)^2+a+b-2}{(a-1)(b-1)} - {\eta_{a,b}}^2\right]$$

where

$$\eta_{a,b} = \frac{(a-b)\sqrt{a+b}}{2}\frac{\Gamma(a-1/2)\Gamma(b-1/2)}{\Gamma(a)\Gamma(b)}$$

1.5 Sinh–arcsinh distribution

Jones and Pewsey (2009) proposed a flexible unimodal four parameter distribution that is induced by sinh–arcsinh (SAS) transformation. The probability density function is

$$p(\theta|\xi,\omega,\epsilon,\delta) = \frac{1}{\omega} f_{SAS}\left(\frac{\theta-\xi}{\omega}\middle|\epsilon,\delta\right)$$

where

$$f_{SAS}(z|\epsilon,\delta) = \frac{1}{\sqrt{2\pi(1+z^2)}}\delta C_{\epsilon,\delta}(z)\exp\left(-\frac{S_{\epsilon,\delta}^2(z)}{2}\right)$$

$$C_{\epsilon,\delta}(z) = \cosh\{\delta\sinh^{-1}(z)-\epsilon\}$$

$$S_{\epsilon,\delta}(z) = \sinh\{\delta\sinh^{-1}(z)-\epsilon\}$$

The SAS distribution can express symmetric and skewed shapes with heavy and light tailweight. ξ and ω are the location and scale parameters, δ (>0) is the kurtosis parameter, and ϵ is the skewness parameter. This distribution is positively (negatively) skewed for $\epsilon > 0$ $(\epsilon < 0)$. The kurtosis is regulated by δ. It can express different shapes with the skew *t*-distributions involving quite sharp and gently sloped ones with various degrees of skewness. The mean and variance of this distribution are

$$\mathrm{E}[\theta] = \xi + \omega\zeta_{\delta,\epsilon}$$

$$\mathrm{V}[\theta] = \omega^2\left[\lambda_{\delta,\epsilon} - \zeta_{\delta,\epsilon}^2\right]$$

where

$$\zeta_{\delta,\epsilon} = \frac{e^{1/4}}{\sqrt{8\pi}}\sinh\left(\frac{\epsilon}{\delta}\right)\left\{K_{(1/\delta+1)/2}\left(\frac{1}{4}\right) + K_{(1/\delta-1)/2}\left(\frac{1}{4}\right)\right\}$$

$$\lambda_{\delta,\epsilon} = \frac{1}{2}\left\{\frac{e^{1/4}}{\sqrt{8\pi}}\cosh\left(\frac{2\epsilon}{\delta}\right)\left\{K_{(2/\delta+1)/2}\left(\frac{1}{4}\right) + K_{(2/\delta-1)/2}\left(\frac{1}{4}\right)\right\} - 1\right\}$$

and $K_a(z)$ is the modified Bessel function of the second kind.

eAppendix 2: Methods for the Bayesian modelling

For the Bayesian random-effects model,

$$Y_i \sim N(\theta_i, \sigma_i^2) \qquad (*)$$
$$\theta_i \sim F(\theta)$$

the prior distributions for the model parameters of $F(\theta)$ directly influence to the posterior inferences and predictions. A representative analysis strategy is to adopt vague prior distributions for all model parameters. In general, the vague prior distributions provide nearly equivalent inference and prediction results with frequentist methods. In **flexmeta** package, we adopted the following vague prior distributions as default settings, and we adopted these prior settings in the applications.

First, for the location and scale parameters ξ and ω, we consistently adopted the following vague prior distributions for the seven models (involving the ordinary normal and *t*-distribution models):

$$\xi \sim N(0, 100^2)$$
$$\omega \sim U(0, 20)$$

For the degree-of-freedom parameter ν of the *t*-distribution, skew *t*-distribution, and AS2 distribution, we adopted an exponential (0.1) prior that was restricted to $\nu > 2.5$ to assure the existence of the second moment ($\nu \geq 2$), as in Fernandez and Steel (1998) and Lee and Thompson (2008). For the skewness parameter α of the skew normal distribution, skew-*t* distribution, and AS2 distribution, we adopted a proper vague normal prior $N(0, 5^2)$. For the Jones–Faddy distribution, we assumed uniform priors for the two model parameters a and b, $a, b \sim U(1.5, 200)$. The lower bound of the uniform distribution is determined to assure the existences of the first, second and third moments (Jones and Faddy, 2003). For the SAS distribution, we also adopted vague priors for the skewness and kurtosis parameters, $\epsilon \sim N(0,100^2)$ and $\delta \sim U(0,100)$.

Using these vague prior distributions, we generally assume a vague prior for the mean μ of the random-effects distribution. For example, the prior of μ for the skew *t*-

distribution is mean: 0.02, SD: 100.4, 2.5th and 97.5th percentiles: −196.7 and 196.8. Note that we also do not involve prior information for the direction of mean parameter μ, because the we assume vague priors for the skewness parameters. For example, for the skew t-distribution example, $\Pr(\mu > 0)$ is 0.50; for the other prior distributions, $\Pr(\mu > 0)$ are nearly 0.50.

Note that the resultant posterior inferences and predictions depend on the prior settings, and the sensitivity should be carefully considered in practices. The source R and Stan codes are available at our GitHub site (https://github.com/nomahi/flexmeta), and users can freely check and customize the prior distribution settings. In addition, informative prior distributions can be adopted by customizing these codes.

References

Azzalini, A. (1985). A class of distributions which includes the normal ones. *Scandinavian Journal of Statistics* **12**, 171-178.

Azzalini, A. (1986). Further results on a class of distributions which includes the normal ones. *Statistica* **46**, 199-208.

Azzalini, A., and Capitanio, A. (2003). Distributions generated by perturbation of symmetry with emphasis on a multivariate skew-*t* distribution. *Journal of the Royal Statistical Society, Series B* **65**, 159-174.

Azzalini, A., and Capitanio, A. (2014). *The Skew-Normal and Related Families*. Cambridge: Cambridge University Press.

Fernandez, C., and Steel, M. F. J. (1998). On Bayesian modelling of fat tails and skewness. *Journal of the American Statistical Association* **93**, 359-371.

Jones, M. C., and Faddy, M. J. (2003). A skew extension of the *t*-distribution, with applications. *Journal of the Royal Statistical Society, Series B* **65**, 159-174.

Jones, M. C., and Pewsey, A. (2009). Sinh-arcsinh distributions. *Biometrika* **96**, 761-780.

Lee, K. J., and Thompson, S. G. (2008). Flexible parametric models for random-effects distributions. *Statistics in Medicine* **27**, 418-434.

Subbotin, M. T. (1923). On the law of frequency of error. *Mathmaticheskii Sbornik* **31**, 296-301.
